# Pretreatment with *Astragalus* polysaccharide alleviates heat stroke–induced intestinal injury in mice

**DOI:** 10.3389/fphar.2025.1612852

**Published:** 2025-09-26

**Authors:** Niqi Shan, Linxiao Wang, Chujun Duan, Yilin Wu, Yangmengjie Jing, Hanyin Fan, Shuai Wang, Yuling Wang, Shijia Wang, Hui Liu, Kun Cheng, Lin Liu, Shanshou Liu, Ran Zhuang

**Affiliations:** ^1^ Department of Immunology, Fourth Military Medical University, Xi’an, Shaanxi, China; ^2^ Department of Emergency, Honghui Hospital, Xi’an Jiaotong University, Xi’an, Shaanxi, China; ^3^ Institute of Medical Research, Northwestern Polytechnical University, Xi’an, Shaanxi, China; ^4^ Department of General Surgery, Tangdu Hospital, Fourth Military Medical University, Xi’an, Shaanxi, China; ^5^ Department of Emergency, Xijing Hospital, Fourth Military Medical University, Xi’an, Shaanxi, China

**Keywords:** Astragalus polysaccharides, heat stroke, intestinal immunity, microbiome, pharmacological network, apoptosis

## Abstract

**Background:**

Heat stroke (HS) is a life-threatening illness. For HS, prevention is more important than treatment. *Astragalus* polysaccharides (APS), a major active ingredient of Astragalus membranaceus (Fisch.) Bunge, has multiple bioactivities, including anti-inflammatory and immunoregulation. This study aimed to evaluate the protective effects of APS on intestinal injury caused by HS.

**Methods:**

Mice were randomized to different groups. After 1 week of APS treatment, a mouse HS model was constructed and evaluated. Intestinal injury was assessed via histopathological examination, and the inflammation level was quantified via quantitative PCR. Flow cytometry and immunofluorescence analyses were used to detect neutrophil infiltration. Gut microbiota was analyzed via 16S rRNA sequencing. Moreover, network pharmacology was employed to analyze the potential targets and functional enrichment of APS. The apoptosis levels were detected in mouse intestinal tissues and IEC-6 intestinal epithelial cells.

**Results:**

APS pretreatment (50 mg/kg BW) prolonged the survival time, delayed the increasing rate of core temperature, and markedly improved organ injuries of HS mice. APS pretreatment improved the pathological changes in the intestine, inhibited inflammation, and reduced neutrophil infiltration. APS enhances the richness of intestinal flora and may shift microbiota functions, thereby benefiting vitamin B metabolism. Network pharmacology analysis indicated the apoptosis pathway as a potential target of APS. *In vivo* experiments using mouse HS model and *in vitro* experiments using IEC-6 cells confirmed the inhibitory effect of APS on apoptosis.

**Conclusion:**

The preventive effects of APS on HS-induced intestinal injury include the alteration of intestinal microbiota composition and anti-inflammatory and antiapoptotic capacity.

## 1 Introduction

In Eastern Asia, the root of *Astragalus membranaceus* (Fisch.) Bunge (AM) has been used as a tonic with health-promoting effects for centuries and is listed as a Chinese medicinal material of the same origin as medicine and food by the National Health Commission in 2023. As documented in *Huangdi Neijing* (Yellow Emperor’s Inner Canon) and *Shennong Ben Cao Jing* (Shennong’s Classic of Materia Medica), the pharmacological properties of *A. membranaceus* (Huang Qi) is warm in nature and nourishes people’s vitality and enriches the blood (Wang et al., 2024). Its benefits also include securing the exterior, disinhibiting water and dispersing swelling, and promoting pus discharge and tissue regeneration Li et al. (2022a). Thus, AM is effective in treating critical illnesses such as infection, trauma, and sepsis (Yu et al., 2022; Ghabeshi et al., 2023). In particular, studies have indicated that *Astragalus* enhances the body’s metabolic rate, boosts small intestinal motility and smooth muscle tone, and facilitates oxidative metabolism within the small intestine (Liu et al., 2022).

*Astragalus* polysaccharides (APS), the principal bioactive constituents extracted from the radix of AM, have been widely studied owing to their therapeutic properties, such as anti-inflammatory, antimicrobial, and antioxidant potentials (Ghabeshi et al., 2023; Fan et al., 2024; Yang et al., 2024). APS has an immune-balancing effect, inhibits the inflammatory response, enhances the immune response, and maintains microcirculatory homeostasis, effectively alleviating organ damage caused by sepsis and minimizing complex shock (Li et al., 2022a). Injectable APS has been widely used in Chinese clinical settings, particularly among chemotherapy patients, with documented positive therapeutic outcomes (Guo et al., 2012). Growing research has increasingly indicated that the diverse therapeutic outcomes of APS observed in various experimental models can be attributed to its ability for modulating intestinal microbiota. For instance, APS has demonstrated significant anti-constipation effects in senescent rat models by restructuring gut microbiome composition and enhancing microbial metabolic pathways (Liu et al., 2022). APS can also exert anti-obesity effects by modulating gut microbial homeostasis in murine models of high-fat diet-induced obesity (Yue et al., 2023). Moreover, regulation of the gut microbiome mediated by APS has been shown to ameliorate polycystic ovary syndrome in murine models (Li et al., 2024b). However, the mechanisms by which APS regulate the gut microbiome remain unclear.

Heat stroke (HS) is the most severe type of heat-related illness and causes high mortality; however, its pathogenesis is complex, and effective therapeutic drugs are lacking (Liu et al., 2020). HS is characterized by a core body temperature (Tc) of >40 °C, and patients with HS present with central nervous system impairment and multiple organ dysfunction syndromes (MODS) (Leon and Bouchama, 2015; Luo et al., 2023a). HS can be classified into classical (nonexertional) and exertional HS based on different susceptible populations; in intensive care units, the mortality rates of the two types of HS can reach 26.5% and 63.2%, respectively (Bouchama et al., 2022). The intestines are particularly vulnerable to heat-induced injury, and the loss of intestinal epithelium can induce a leaky gut as the translocation of gut microbiota and bacterial toxins into circulation, which contributes to systemic inflammation and consequently MODS (Shih et al., 2023). Gut barrier permeability is modulated through a multifactorial interplay involving environmental stimuli, epithelial apoptotic pathways, cytokine networks, and immunocyte activity (Jia et al., 2023).

Previous studies have reported that the pathogenesis of HS is closely associated with inflammation and stress, and compounds derived from AM can confer protection against HS. Calycosin-7-O-β-D-glucoside, a calycosin derivative compound extracted from AM, reduced myocardial injury in rats with HS (Tsai et al., 2019). Furthermore, astragaloside-IV (AS-IV) alleviated heat-induced inflammation by inhibiting endoplasmic reticulum stress and autophagy (Dong et al., 2017b) and heat-induced apoptosis by inhibiting the excessive activation of the mitochondrial Ca^2+^ uniporter (Dong et al., 2017a). However, the role of APS in critical diseases remains largely unknown.

Based on these findings, the current study sought to investigate the potential effects of APS on a mouse model of HS and an LPS-induced sepsis-like model with IEC-6 cells, a well-established intestinal epithelial cell line commonly employed *in vitro* to study mechanisms of intestinal barrier function (Jia et al., 2023; Sampath et al., 2024). This approach was intended to further elucidate the role of gut microbiota throughout the process and the potential mechanisms involved.

## 2 Materials and methods

### 2.1 Mice

Male C57BL/6J mice (10–12-week-old with body weight of 24 ± 2 g) were purchased from the Animal Center of the Fourth Military Medical University. All mice were housed under specific pathogen-free conditions and fed standard animal chow and water. The animal experiments were approved by the Ethics Committee of the Fourth Military Medical University (license no. 20211016).

### 2.2 Preparation of the HS model

The mice were exposed to a controlled climate chamber (ambient temperature: 41.0 °C ± 0.5 °C, humidity: 60% ± 5%; XinRuan, Shanghai, China) without water and food. The rectal temperature was monitored as the core temperature (Tc) using a digital thermometer (BW-TH1101, Billion, Shanghai, China) every 15 min. When the Tc reached or exceeded 42.0 °C, the mice exhibited hind limb weakness and impaired locomotion, indicating the onset of HS. Then, the mice were maintained at room temperature (25.0 °C ± 0.5 °C) to recover until they were sacrificed (Leon et al., 2005; Luo et al., 2023a; Ji et al., 2024). Routine blood tests were performed using a DF-3000Vet automatic animal blood cell analyzer (Beijing, China).

### 2.3 Drug administration

The mice were randomly assigned to control (CTR) (n = 14), HS + normal saline (NS) (n = 15), and HS + APS (25, 50, 100, and 200 mg/kg BW; n = 14, 16, 16, and 12, respectively) groups. APS was administered intraperitoneally, and the concentrations were selected based on previous studies to ensure an appropriate balance between safety and therapeutic efficacy (Dong et al., 2019; Zhang et al., 2020; Ding et al., 2021; Ming et al., 2022; Li et al., 2023; Wei et al., 2023). After 1 week of treatment (once daily via intraperitoneal injection), the mice were exposed to heat stress, except for the CTR mice. Highly purified APS (purity exceeding 90%) was purchased from Tianjin Cinorch Pharmaceutical Co., Ltd. (Lot# 221205, China), with an average molecular weight ranging from 20,000 to 60,000 Da (patent no. ZL00811547.8). APS was purified using the water-extraction and alcohol-precipitation technique (Zhou et al., 2013). First, the root of AM was soaked and decocted in the stilled boiling water for extraction. After vacuum condensation, the liquid extract was precipitated and washed with various concentrations of alcohols to obtain the crude product (i.e., bound polysaccharides). This crude product then underwent filtration, elution, and ultrafiltration. The concentrated supernatant was further precipitated and washed with alcohols. Finally, the precipitates were lyophilized for analysis and experiments, yielding purified APS as a white powder suitable for human injection, which has been approved under the National Medical Products Administration of China. APS was detected using size-exclusion chromatography with refractive index detector (SEC-RID). The plant name (AM) had been confirmed through http://www.theplantlist.org.

### 2.4 Histological analysis

Blood samples were collected using two methods: (1) medial canthus vein puncture before modeling and (2) terminal puncture via ocular enucleation at 3 h after HS that rectal temperature reached its lowest point followed by cervical dislocation (Li et al., 2021). All procedures were performed under isoflurane inhalational anesthesia and in compliance with the guidelines of the Institutional Animal Care and Use Committee. After cervical dislocation, the tissues were obtained and fixed with 4% paraformaldehyde. Subsequently, the tissues were embedded in paraffin and processed into 5-µm thick sections. Routine hematoxylin and eosin (H&E) staining was performed, and the specimens were imaged under a light microscope using a digital camera system (SlideView VS200, Olympus, Tokyo, Japan). Assessment of pathological changes in the intestines was blindly performed using the Olympus OlyVIA system (Tokyo, Japan). The villus height and crypt depth of the duodenal, jejunal, and ileal tissues were measured in six randomly selected fields of each slide. The crypt depth and muscle thickness in the colon tissue were measured in 10 random fields.

### 2.5 Flow cytometry (FCM) analysis

Intestinal lamina propria (LP) cells from mice were isolated using our previously described protocol (Wang et al., 2023). For FCM, the cells were incubated with anti-mouse CD16/CD32 Abs (BD Biosciences, San Jose, CA, United States) for 20 min at 4 °C, and then fluorochrome-labeled antibodies (eBioscience, Thermo Fisher Scientific) were added in a total volume of 100 μL, mixed thoroughly, and incubated for 30 min at 4 °C. FCM analysis was performed using an SA3800 spectral cell analyzer (Sony Biotechnology, Tokyo, Japan).

### 2.6 Immunofluorescence

To examine neutrophils, paraffin-embedded sections were prepared following the standard protocol and then incubated for 2 h at room temperature with mouse monoclonal antibody against myeloperoxidase (MPO; GB15224, Servicebio, China). Next, these specimens were incubated with a Cy3-labeled goat anti-mouse secondary antibody. The nuclei were counterstained with 4′,6-diamidino-2-phenylindole (DAPI) and then visualized under a fluorescence microscope (Olympus).

### 2.7 Quantitative polymerase chain reaction (qPCR)

Total RNA was extracted using TRIGene reagent (GenStar, China) and converted to cDNA using the Hifair Ⅱ 1st Strand cDNA Synthesis Kit (Yeasen Biotechnology, Shanghai, China). qPCR analysis was performed using the SYBR Master Mix (GenStar), and the results were quantified via the 2^−ΔΔCT^ method. β-actin was used as the internal control. Transcript levels were normalized to transcript levels of β-actin.

### 2.8 The 16S ribosomal ribonucleic acid (16S rRNA) sequencing

To avoid the cage effect, the mice were randomized to treatment groups within a cage before the first injection. Freshly collected feces were obtained and stored immediately at −80 °C. The 16S rRNA sequencing of the gut microbiota was performed using high-throughput sequencing (Genedenovo Biotechnology Ltd., Guangzhou, China). There were six samples in each group.

### 2.9 Detection of vitamin B6

To assess the serum vitamin B level, a chemiluminescence assay kit was used (Keming Biotechnology, Suzhou, China). Briefly, the sample, standard solution, and reagents were added following the manufacturer’s protocol, mixed thoroughly, and reacted at 25 °C for 20 min. The absorbance at 390 nm was measured using a microplate reader (Infinite200 PRO, Tecan, Männedorf, Switzerland), and the ΔA value and vitamin B6 content were calculated based on the standard curve.

### 2.10 Network pharmacology analysis

The main components of APS are D-glucose, glucuronic acid, galacturonic acid, hexuronic acid, rhamnose, and arabinose (Xu et al., 2024b). The corresponding SMILES expression levels were obtained from PubChem (https://pubchem.ncbi.nlm.nih.gov/). The potential target genes of these six main components were predicted by three target prediction databases with corresponding screening conditions: SwissTarget Prediction Database (http://swisstargetprediction.ch/, screening parameter: probability of >0, species: *Homo sapiens*), SuperPred database (http://prediction.charite.de/, screening parameter: model accuracy >80%), and TargetNet database (http://targetnet.scbdd.com/, screening parameter: probability of >0). HS-related genes were obtained from GeneCards (https://www.genecards.org/) and OMIM (https://omim.org/) databases by entering “heat stroke” as a keyword. The potential target genes predicted by each APS component were intersected with all HS-related genes to obtain common genes, which are the putative therapeutic targets. The protein–protein interaction (PPI) network was constructed using the String (https://cn.string-db.org/) database (the organism was selected as human and the score was set to high confidence of 0.7). The DAVID functional annotation tool (https://david.ncifcrf.gov/) was used to perform Gene Ontology (GO) and Kyoto Encyclopedia of Genes and Genomes (KEGG) functional enrichment analyses.

### 2.11 *In situ* detection of apoptotic cells

Apoptotic cells in the small intestinal tissue were detected using the TUNEL assay, following the manufacturer’s protocol (Thermo Fisher Scientific). Apoptotic cells were imaged under an Olympus microscope. The TUNEL-positive cells were counted in 10 randomly selected high-power fields at 400× magnification.

### 2.12 Western blot analysis

Protein samples were prepared using ristocetin-induced platelet aggregation lysate and a protease inhibitor, separated through sodium dodecyl sulfate–polyacrylamide gel electrophoresis, and transferred to polyvinylidene fluoride membranes. Proteins were detected using the enhanced chemiluminescence reagent and Chemidoc imaging system (Bio-Rad) following incubation with primary and secondary antibodies. The following primary antibodies were obtained from Cell Signaling Technology (Massachusetts, United States) and Proteintech (Chicago, United States): caspase-3, cleaved caspase-3, caspase-9, cleaved caspase-9, Bax, Bak, ZO-1, and occludin. The expression level of the target protein was quantified as the ratio of the gray intensity to that of β-actin.

### 2.13 Cell culture

IEC-6 cells (originated from rat small intestinal crypts) were purchased from ATCC and maintained in Dulbecco’s modified Eagle medium containing 10% fetal bovine serum. Cells were treated with different doses of APS (100, 200, 400 μg/mL) 1 h before challenging with lipopolysaccharide (LPS; 10 μg/mL), followed by a 24-h cultivation period. Each experiment was replicated in at least three independent parallel tests.

### 2.14 Live/dead cell staining

Cells were collected and washed with assay buffer, incubated with Calcein-AM and propidium iodide (PI) solutions following the manufacturer’s protocol (Solarbio, Beijing, China), and photographed under a fluorescence microscope (EVOS, Life Technology). The percentage of live (Calcein-AM^+^PI^−^) cells was calculated.

### 2.15 Cell counting kit-8 (CCK-8) assay

Cell viability was measured using the CCK-8 assay. Briefly, cells were seeded into 96-well plates and treated with LPS with or without APS for 24 h. Then, 10 μL of the CCK-8 reagent was added to each well. The plate was incubated at 37 °C for 4 h. The absorbance was recorded at 450 nm using a multiscan FC microplate reader (Thermo Scientific).

### 2.16 Statistical analysis

Statistical data were analyzed using GraphPad Prism 9.0 (GraphPad, La Jolla, CA, United States). Survival curves were plotted using the Kaplan-Meier method and compared using the log-rank test. Student’s t-test or one-way analysis of variance was used to compare different groups. *P*-values of <0.05 were considered to indicate statistical significance.

## 3 Results

### 3.1 Pretreatment with APS improves heat tolerance in mice

**FIGURE 1:**
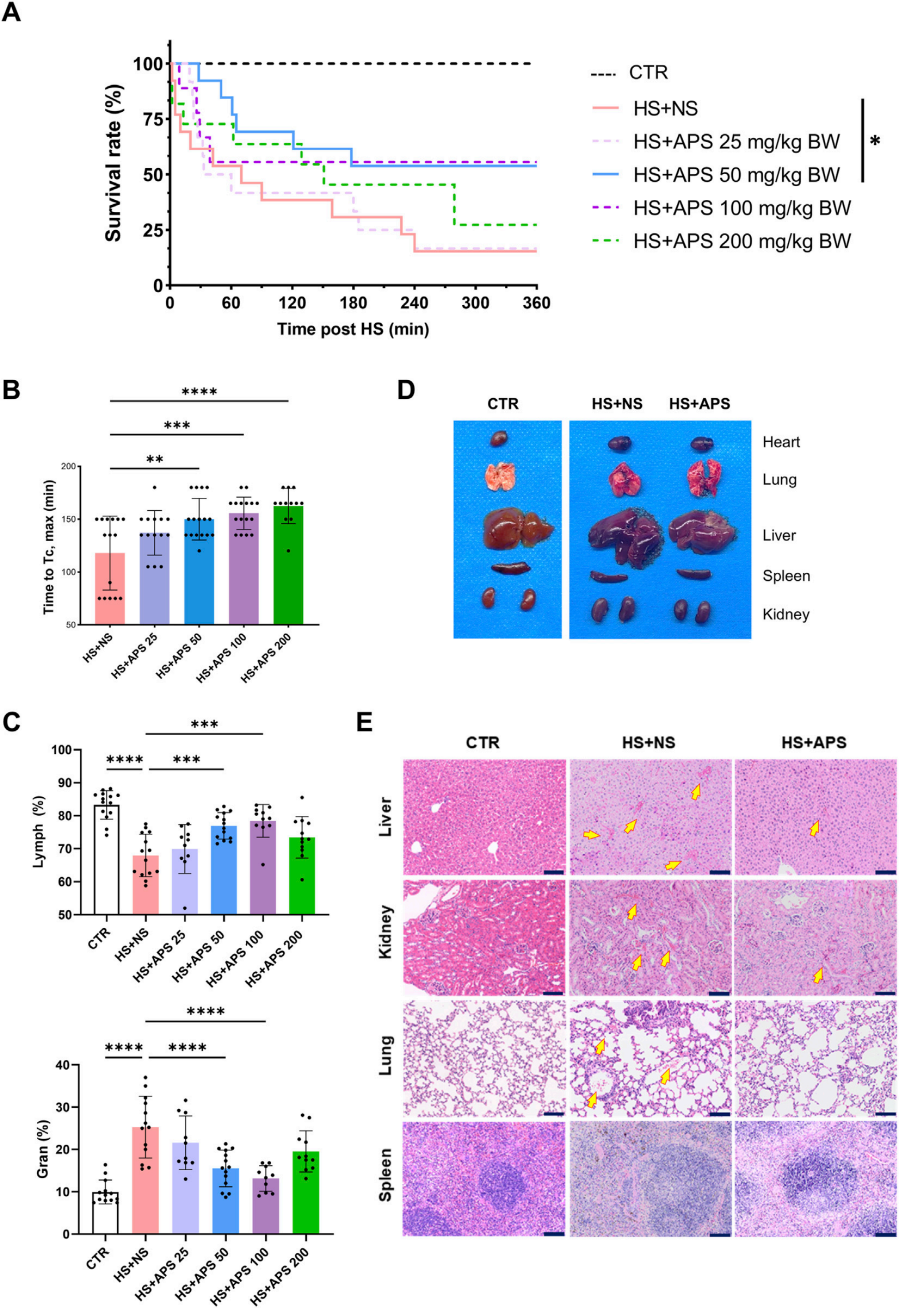
Pretreatment with APS alleviates injuries and improves resistance to HS. **(A)** Survival time after HS pretreated with NS or different doses of APS. **(B)** APS delayed the increase in core temperature (Tc). **(C)** Routine blood test at 3 h post-HS. Percentage of lymphocytes (Lymph %) and granulocytes (Gran %) in the whole blood across groups. **(D)** Photos of murine tissues (including the heart, lungs, livers, spleen, and kidneys) in different groups. Pretreatment with 50 mg/kg BW APS was adopted. **(E)** The tissues of the mice were photographed after H&E staining. Tissues were harvested at 3 h after HS onset (Scale bar = 100 μm). Arrows point to areas of tissue hemorrhage. N = 3 for histopathological experiments. Congestion Pretreatment with 50 mg/kg BW APS was adopted. Each dot indicates the values of individual mice. Data were presented as mean ± standard deviation; *P < 0.05, **P < 0.01, ***P < 0.001, ****P < 0.0001.

First, the mouse model of HS was established. Although different doses of APS pretreatment can improve the survival rate of HS mice compared with the HS + NS mice, only 50 mg/kg BW APS pretreatment group showed a significant difference (Figure 1A). Compared with the HS + NS group, the increasing rate of Tc in the HS + APS groups was slowed down, evidenced by the time to Tc max of the mice that was significantly longer in the HS + APS (>50 mg/kg BW) groups than in the HS + NS group (Figure 1B).

Regarding cell proportion, HS exposure can significantly reduce the proportion of lymphocytes and increase the proportion of granulocytes in the blood, whereas 50 and 100 mg/kg doses of APS pretreatment can reverse these effects (Figure 1C). Collectively, pretreatment with 50 mg/kg BW of APS had the best effects and was used for follow-up experiments.

APS improves HS-induced damage to organs. As shown in Figure 1D, multiple organ injuries developed during HS. The organs in the HS + APS group were more swollen, darker in color, and even had more bleeding points than were those in the HS + NS group. Further histopathological examination of various organs indicated that APS treatment reduced the risk of HS onset in mice. Specifically, the HS + NS group had damaged liver lobules and abnormally congested hepatic sinusoids and venules. The disruption of some glomerular structures was accompanied with congested interstitial blood vessels and lymphocyte infiltration; the alveolar wall capillaries showed signs of dilation and congestion, with hemorrhage in the alveolar cavities and lymphocyte infiltration. Moreover, alterations were noted in the spleen, where the structures of both white and red pulps were disturbed with unclear boundaries; all these injuries were ameliorated in the mice pretreated with APS (Figure 1E).

### 3.2 APS mitigates HS-induced intestinal damage

**FIGURE 2:**
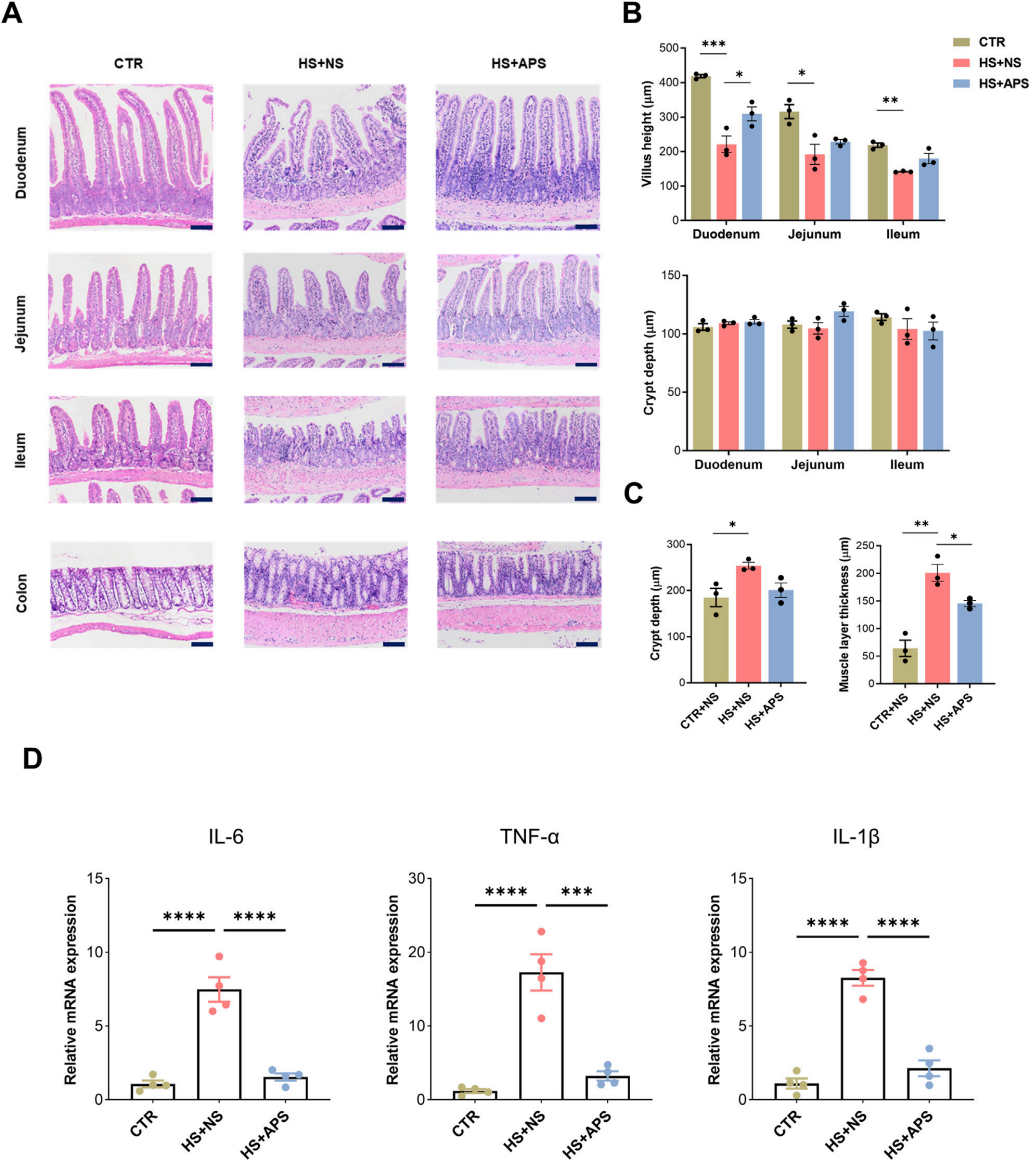
Beneficial effects of APS on the intestine. **(A)** Representative image of intestinal H&E showing that 50 mg/kg BW APS partially restored HS-induced mucosal damage. Detachment of the intestinal villi tips was mainly observed in the duodenum and ileum tissues. **(B)** Statistics of villus length and crypt depth in the small intestine. **(C)** Statistical analysis of the depth of the colonic crypts and muscle layer thickness in the colon. The villus height and crypt depth measurements of the villi and crypts per animal were assessed in at least six randomly selected fields (per mouse) (Scale bar = 100 μm). **(D)** qPCR analysis for inflammation cytokines of the intestines (n = 4). Each dot indicates the values of individual mice. Data were presented as mean ± standard error of the mean; *P < 0.05, **P < 0.01, ***P < 0.001, ****P < 0.0001.

Next, we assessed the intestines histologically; H&E staining revealed that HS induced the loss of the intestinal epithelium and damage occurred in both the small and large intestines. In the HS + NS group, the villi in the small intestines (duodenum, jejunum, and ileum) were remarkably shortened, but the depth of the crypts was not altered. In most of the mice, the intestinal villi tips were detached. In addition, it is accompanied with enhanced inflammatory cell infiltration in the LP. However, APS attenuated the pathological damage to the small intestines in HS mice (Figures 2A,B). Meanwhile, increased depth of the crypts and a thickened mucus layer were observed in the colons. Similar to the small intestine, APS also attenuated the pathological damage in the large intestines of HS mice (Figures 2A,C).

Inflammatory cytokines were measured at the time of sample collection to test the efficacy of APS on the intestines. The mRNA levels of IL-6, TNF-α, and IL-1β were elevated following HS exposure, whereas APS pretreatment showed marked inhibition of inflammatory cytokines, indicating marked alterations in the intestinal inflammation of the mice (Figure 2D).

### 3.3 APS improves intestinal immunity in HS mice

**FIGURE 3:**
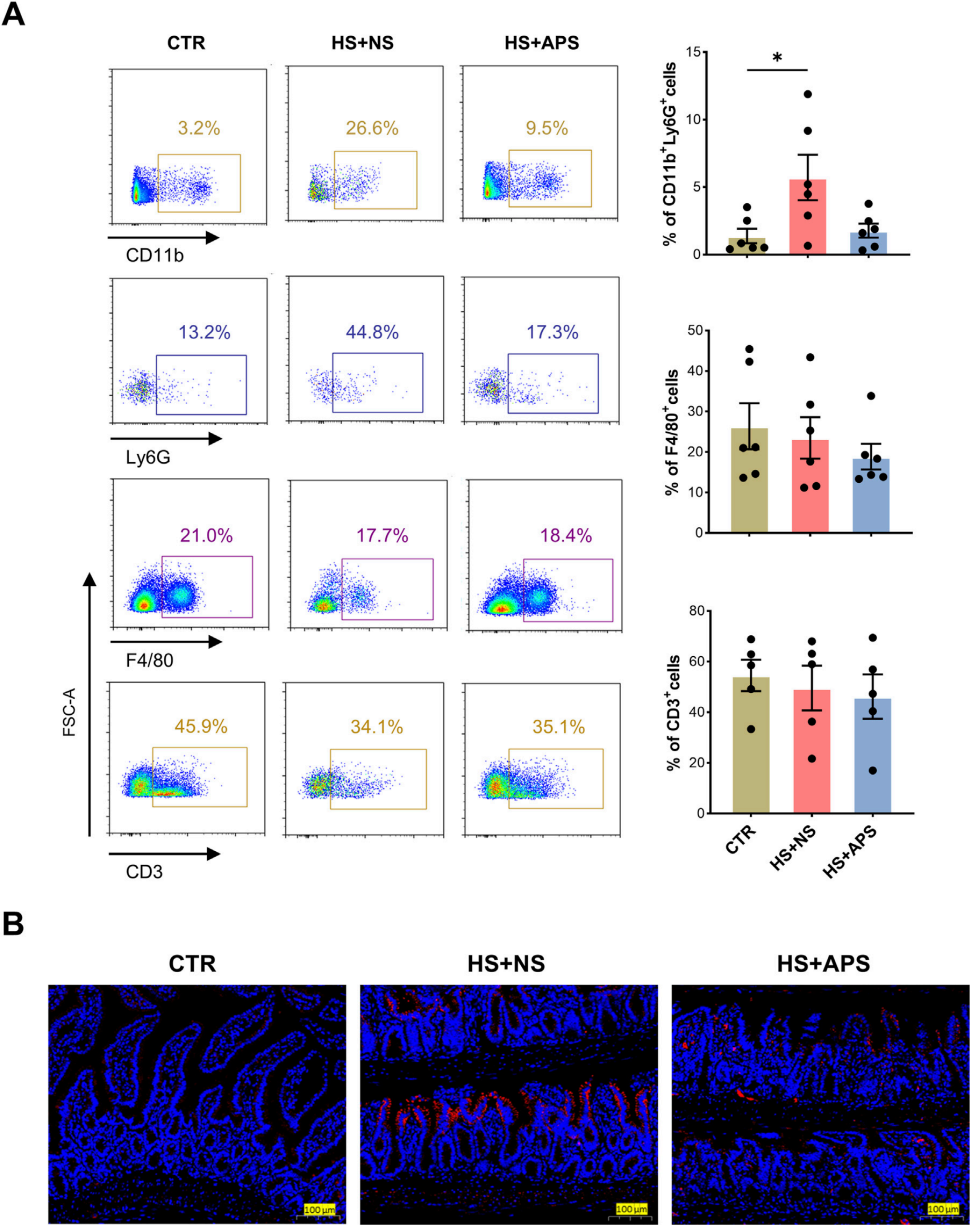
APS improved intestinal immunity. **(A)** FCM analysis for the percentage of neutrophils (CD45^+^CD11b^+^Ly6G^+^), macrophages (CD45^+^F4/80^+^), and T cells (CD45^+^ CD3^+^) in the LP of the small intestine (n = 4-6). The LP cells were pre-gated on the CD45^+^ leukocyte population. **(B)** Immunofluorescence staining for MPO to detect neutrophil infiltration (Scale bar = 100 μm). Pretreatment with 50 mg/kg BW APS was adopted. Each dot indicates the values of individual mice. Data were presented as mean ± standard error of the mean; *P < 0.05.

Neutrophil infiltration was assessed through FCM analysis and immunofluorescence staining of MPO^+^ cells, as an overexuberant neutrophil response is known to cause epithelial damage in the inflammatory intestine (Williams and Parkos, 2007). As shown in Figure 3A, compared with the CTR mice, HS + NS mice had a higher proportion of neutrophils, and APS showed marked inhibition of neutrophils; however, the proportion of macrophages and T lymphocytes remained unchanged. These results indicated that in the early/acute phase of HS, neutrophils are first-line defenders, and their dysfunction inducing the innate immune inflammatory response leads to mortality and poor outcomes. Additionally, the LP of the ileal region was rich in immune cells, and the HS + NS mice had a higher number of MPO^+^ cells than the CTR mice, with the HS + APS group showing significantly less neutrophil infiltration than the HS + NS group (Figure 3B).

### 3.4 APS alters the microbiota composition

Modern pharmacological studies have shown that APS exerts favorable effects on organ damage. However, the role of APS in gastrointestinal tract and their underlying protective mechanisms remain unclear. Therefore, we aimed to determine the role of APS in the gut from the perspective of microbial transformation.

**FIGURE 4:**
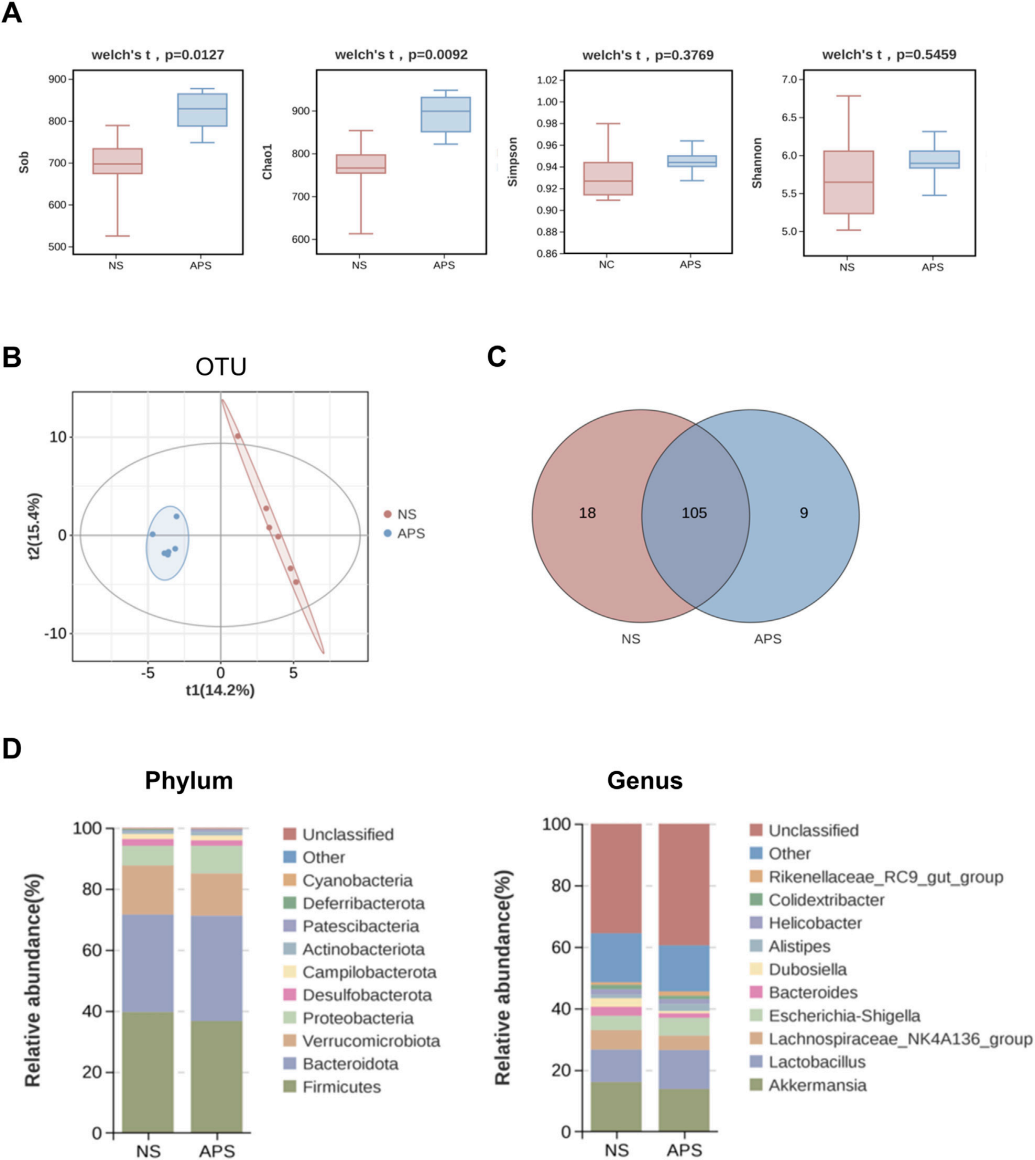
Alteration in gut microbiota composition due to APS by 16S rRNA sequencing (n = 6 per group). **(A)** α-Diversity of gut microbiota. **(B)** β-Diversity represented by a PLS-DA plot at the operational taxonomic unit (OTU) level. **(C)** Venn diagram of overlapping and exclusive genera in the NC and APS-administered groups. **(D)** Abundance bar plot at the phylum level (left); abundance bar plot at the genus level (right).

After read-quality filtering, denoising, and clustering, high-quality bacterial 16S rRNA gene sequences were successfully obtained from 12 samples. The gut microbial abundance curve revealed that the sample in each group had sufficient microbial abundance (data not shown). Gut microbiota was assessed using α-diversity indexes, with the Sob and Chao1 indexes reflecting the community richness and the Shannon and Simpson indexes reflecting the community diversity. As shown in Figure 4A, the Sob and Chao1 indexes of the APS group were significantly higher than those of the NS control group, whereas the Shannon and Simpson indexes showed no differences between the two groups, indicating that APS had a significant effect on microbiota richness but no effect on microbiota evenness.

Next, we compared the microbial community similarity using two β-diversity measures: Partial Least Squares Discrimination Analysis (PLS-DA) and Anosim. PLS-DA scatterplots are depicted in Figure 4B, which revealed that APS was significantly different from NC at the operational taxonomic unit level, indicating that the microbial community was distinctly altered by APS administration. Anosim analysis confirmed a significant difference between the two groups (*P* = 0.019).

As shown in Figure 4C, the Venn plot demonstrated that 105 genera coexisted in the two groups and nine genera were unique to the APS group, including *Frisingicoccus*, Gram-negative bacterium cTPY-13, Methylobacterium–Methylorubrum, *Lactococcus*, *Microbulbifer*, *Nosocomiicoccus*, *Facklamia*, *Candidatus*, *Paracaedibacter*, and *Fusobacterium*. The detailed results of cluster analysis of the intestinal flora are shown in Figure 4D. Firmicutes, Bacteroidetes, Verrucomicrobia, Proteobacteria, Desulfobacterota, Campilobacterota, and Actinobacteriota were the most dominant phyla in each group. In terms of abundance, the top three flora at the genus level were *Akkermansia*, *Lactobacillus*, and Lachnospiraceae_NK4A136_group.

### 3.5 APS modulates gut microbiota function and vitamin B6 metabolism

**FIGURE 5:**
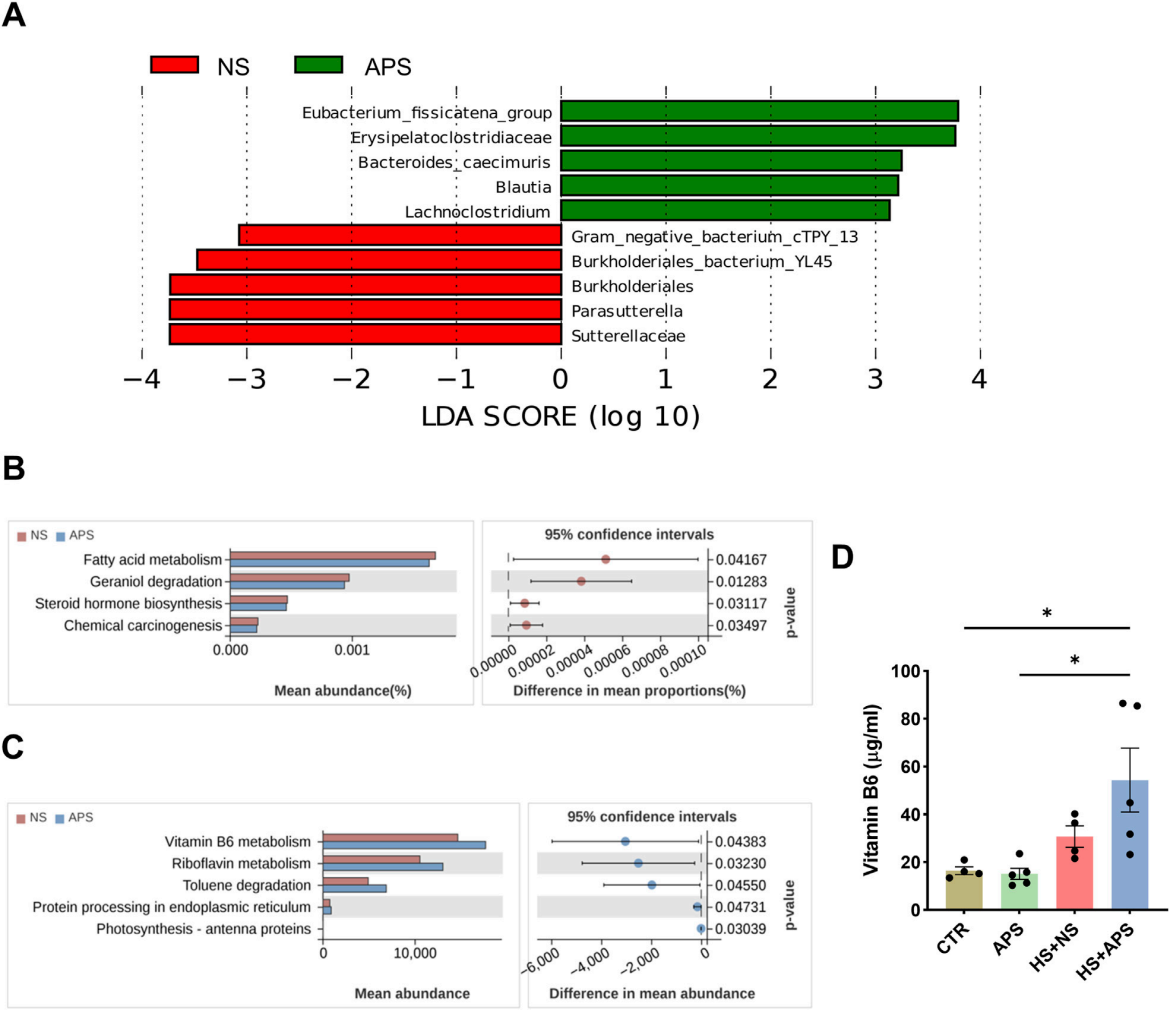
Alteration in gut microbiota function due to APS. **(A)** Linear discriminant analysis (LDA) combined with effect-size measurements at all levels of the NC and APS-administered groups. LDA scores that are greater in the APS group are shown in red, whereas the scores that are elevated in the NC group are depicted in green. **(B)** Predicted bacterial functions using Tax4Fun analysis at KEGG level 3 pathways. **(C)** Predicted bacterial functions using PICRUSt2 analysis at KEGG level 3 pathways. **(D)** Vitamin B6 levels in the serum (n = 4-5). Each dot indicates the values of individual mice. Data were presented as mean ± standard error of the mean; *P < 0.05.

Linear discriminant analysis (LDA) effect size (LEfSe) was used to screen specific species to further determine the effects of APS on intestinal flora. As shown in Figure 5A, compared with the NS group, the relative abundances of some genera, including eubacterium_fissicatena_group, Erysipelatoclostridiaceae, *Bacteroides*_caecimuris, Blautia, and Lachnoclostridium, significantly reduced, whereas the abundances of Gram_negative_bacterium_cTPY_13, Burkholderiales_bacterium_YL45, Burkholderiales, Parasutterella, and Sutterellaceae evidently increased in the APS group (*P* < 0.05, LDA score >3).

Bacterial functions were predicted using PICRUSt2 and Tax4Fun algorithms. Tax4Fun analysis showed that the functional categories of fatty acid metabolism, geraniol degradation, steroid hormone biosynthesis, and chemical carcinogenesis were significantly different between NS and APS groups (Figure 5B). PICRUSt2 analysis revealed that compared with the NC group, APS-induced bacterial functional changes in KEGG level 3 were mainly involved in vitamin B6 metabolism, riboflavin metabolism, toluene degradation, and protein processing in the endoplasmic reticulum (Figure 5C). Systemic vitamin B6 deficiency has previously been associated with increased oxidative stress and inflammation (Lysne et al., 2016). Riboflavin is a natural antioxidant, and the depletion of riboflavin causes endoplasmic reticulum stress (Zhang et al., 2022a; Zhang et al., 2022b). Moreover, vitamin B6 and riboflavin may play important protective roles against heat stress at the cellular level (Sammad et al., 2022; Zhang et al., 2022b). These results indicated that the impact of APS on the microbiota structure was also accompanied with changes in microbial function.

Vitamin B6 deficiency has been reported to contribute to inflammatory disease, and vitamin B6 supplementation can reverse these effects in deficiency states. To verify the effect of APS on vitamin B6, we detected its level in the serum. In the resting state, APS did not significantly affect vitamin B6 levels. Within 3-h post-HS, vitamin B6 levels showed a compensable elevated trend. We then assessed the serum levels of vitamin B6 in the HS group pretreated with APS. However, compared with the CTR and APS groups, APS notably elevated serum vitamin B6 levels after HS (Figure 5D).

### 3.6 Identification of potential targets against HS

**FIGURE 6:**
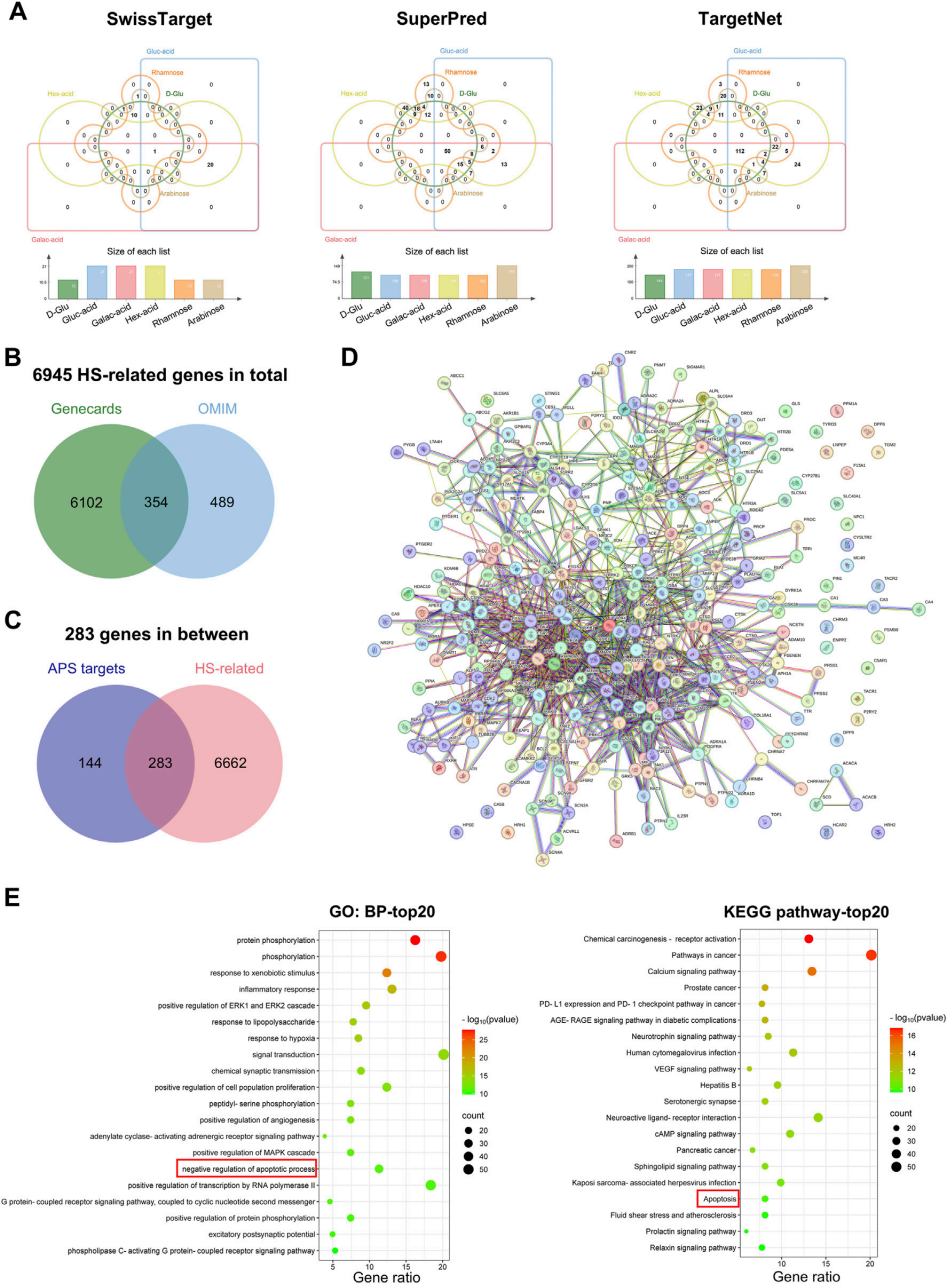
Pharmacological network. Identification of potential targets. **(A)** Venn diagram of the potential target genes of the six major components of APS (D-Glu: D-Glucose, Gluc-acid: Glucuronic acid, Galac-acid: Galacturonic acid, Hex-acid: Hexuronic acid, Rhamnose, and Arabinose) in the SwissTarget, SuperPred, and TargetNet databases. The Venn diagram was constructed using the online jvenn tool. **(B)** Venn diagram of HS-related genes from GeneCards and OMIM databases. **(C)** Venn diagram of all HS-related genes and APS-related targets. **(D)** PPI network of the 283 common genes from String databases. **(E)** Top 20 enriched pathways from GO-biological process (BP) and KEGG functional enrichment analyses of the 283 common targets.

To further explore the underlying mechanism of APS treatment for HS, we used three target prediction databases to identify and analyze the potential target genes of the six major components of APS (D-glucose, glucuronic acid, galacturonic acid, hexuronic acid, rhamnose, and arabinose). A total of 33 potential targets were obtained in the SwissTarget database, 212 in the SuperPred database, and 248 in the TargetNet (Figure 6A). After deleting duplicate targets, a final list of 427 potential APS-related targets was obtained. In addition, 6456 HS-related genes were retrieved from the GeneCards database and 843 from the OMIM database, yielding a total of 6945 HS-related genes after removing duplicates (Figure 6B). The intersection of APS potential target genes and HS-related genes was then calculated, revealing 283 common genes, which were considered potential therapeutic targets of APS for HS treatment (Figure 6C). These target genes were next imported into the String database to construct a PPI network (Figure 6D) and underwent GO and KEGG functional enrichment analyses. The results demonstrated that these target genes were enriched in pathways such as phosphorylation, inflammatory response, positive regulation of ERK1 and ERK2 cascade, response to LPS, and response to hypoxia. Notably, the apoptosis-related pathway was enriched in both GO and KEGG analyses (Figure 6E).

### 3.7 APS maintains tissue homeostasis by regulating apoptosis

**FIGURE 7:**
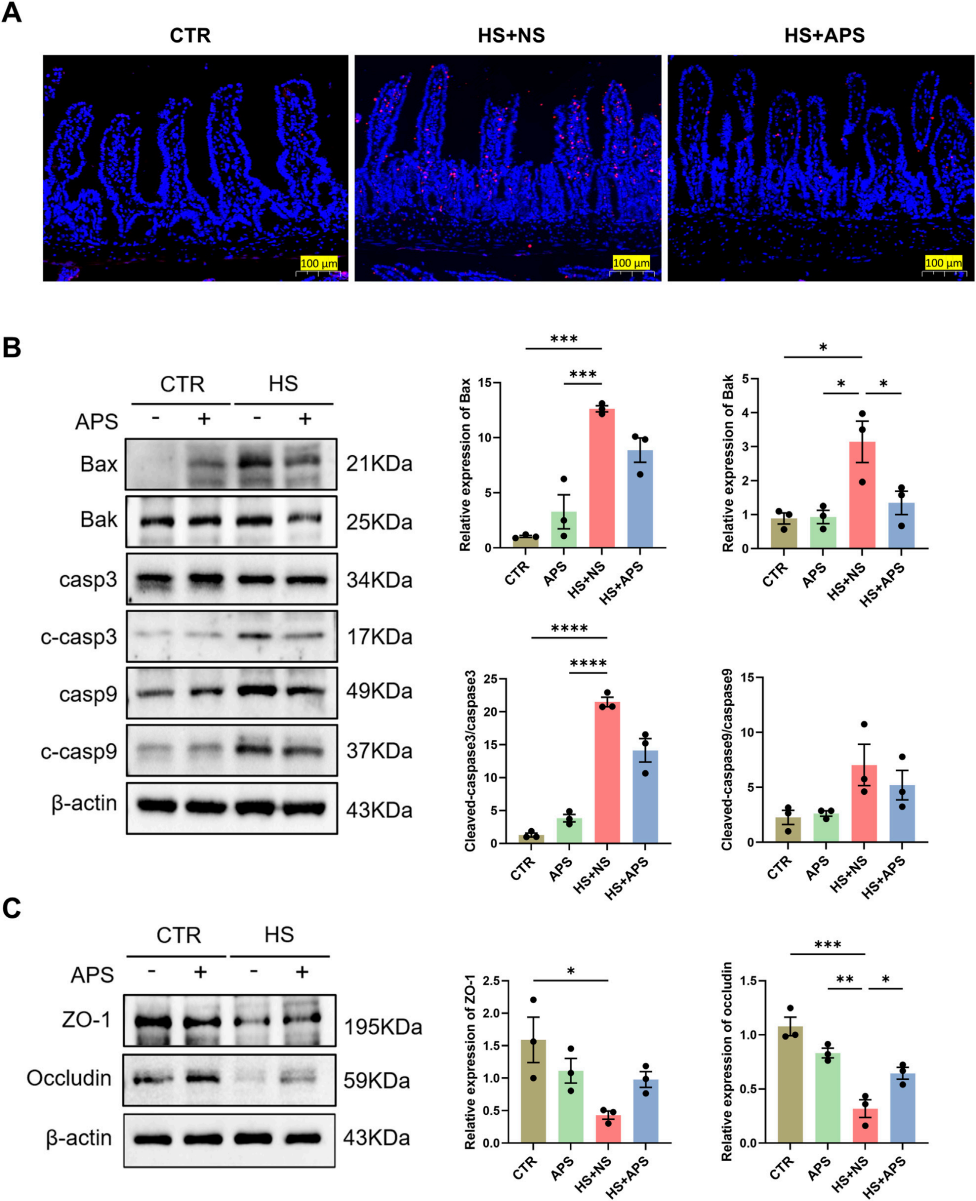
Pretreatment with APS (50 mg/kg BW) decreased intestinal apoptosis induced by HS in mice (n = 3 per group). **(A)** TUNEL staining. Positive cells are seen as red fluorescence with DAPI used as a counterstain (Scale bar = 100 μm). **(B)** The expression of apoptotic proteins in the intestine was detected by Western blot. β-actin was used as a loading control. **(C)** The expression of tight junction proteins ZO-1 and occludin in the intestine was detected and quantitatively compared among the groups. Data were presented as mean ± standard error of the mean; *P < 0.05, **P < 0.01, ***P < 0.001, ****P < 0.0001.

Dysfunctions in apoptosis are implicated in HS pathogenesis. We used the TUNEL assay to detect apoptotic DNA fragmentation in the intestine. HS mice showed more cells positive for apoptotic DNA fragmentation than the NC mice; however, there were significantly fewer positive cells in the HS + APS mice compared with those in the HS + NS mice (Figure 7A).

The effects of APS on apoptotic pathways were further assessed via Western blot analysis. The results demonstrated a marked increase in the protein levels of Bax, Bak, and cleaved caspase-3 following HS exposure. Notably, pretreatment with APS significantly attenuated the HS-induced upregulation of intrinsic apoptotic proteins, as illustrated in Figure 7B. Concurrently, we observed a reduction in the expression of the tight junction proteins zonula occludens (ZO)-1 and occludin in the HS + NS group, indicating a compromised intestinal barrier integrity. However, this impairment was mitigated in the HS + APS group (Figure 7C). These findings suggest that APS has therapeutic potential in mitigating the intestinal damage caused by HS by modulating the apoptotic process.

**FIGURE 8:**
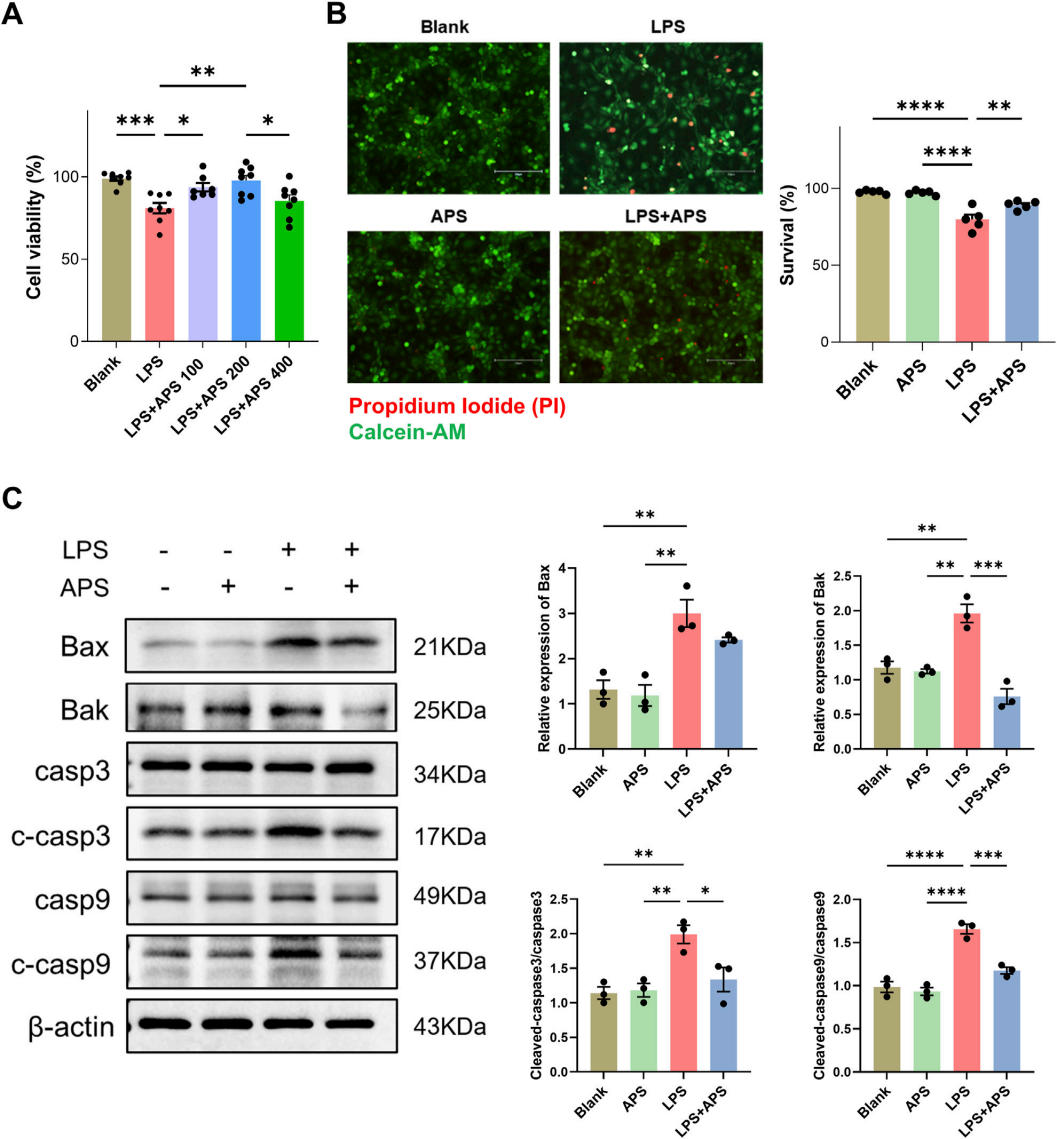
APS decreased apoptosis induced by LPS in IEC-6 cells. **(A)** IEC-6 cells were treated with LPS and different doses of APS for 24 h and then subjected to the cell viability CCK-8 assay (n = 8). **(B)** Representative images of Calcein-AM/PI staining and quantification of the percentages of live cells (Calcein-AM^+^PI^−^) among groups (n = 5). Treatment with 200 μg/mL APS was adopted. Positive staining of Calcein-AM indicates live cells and PI indicates dead cells (Scale bar = 150 µm). **(C)** The expression of apoptotic proteins in IEC-6 cells was detected by Western blot (n = 3). Treatment with 200 μg/mL APS was adopted. β-actin was used as a loading control. Data were presented as mean ± standard error of the mean; *P < 0.05, **P < 0.01, ***P < 0.001, ****P < 0.0001.

Similar results were observed in IEC-6 intestinal epithelial cells. We first performed CCK-8 assay to evaluate the viability of cells treated with different doses of APS, and found that treatment with 200 μg/mL APS exhibited the best cell viability (Figure 8A). We then employed this dose of APS to conduct further Calcein-AM/PI staining. Results revealed a significant decrease in live cells after LPS treatment, and treatment with APS significantly mitigated the LPS-induced upregulation of cell death (Figure 8B). Western blot analysis demonstrated a substantial increase in the protein levels of Bax, Bak, cleaved caspase-3, and cleaved caspase-9 following LPS stimulation; however, APS treatment significantly attenuated the LPS-induced upregulation of these apoptotic proteins, as shown in Figure 8C.

## 4 Discussion

The current study showed that pretreatment with APS could potentially treat HS and its complications. Our results revealed that APS can enhance heat tolerance in mice, indicating that it exerts a protective effect against physiological stress induced by elevated temperatures.

The slower rate of increase in core temperature and prolonged duration to reach the maximum temperature observed in the HS + APS group suggest that APS administration serves as a prophylactic treatment to delay the onset of heat stress symptoms. Furthermore, the protective effect of APS on organ systems is a critical consideration. The gastrointestinal system is highly susceptible to heat-induced injury during HS (Leon and Helwig, 2010; Lian et al., 2020). Disruption of the intestinal barrier has emerged as a critical and contributor to HS (Sun et al., 2024). Currently, a “sepsis-like” mechanism has been widely accepted, indicating that heat exposure damages the vascular endothelial and intestinal barrier. This damage then causes the dysregulation of intestinal microbiota and penetration of endotoxins (e.g., LPS) as pathogenic bacteria into the systemic circulation (referred to as “leaky gut”), consequently triggering the overproduction of pro-inflammatory cytokines, culminating in a life-threatening cytokine storm and ultimately resulting in systemic inflammatory response syndrome and MODS (Xia et al., 2017; Lim, 2018; Ogden et al., 2020; Luo et al., 2023b). Meanwhile, this cooperative interaction further drives the pathogenesis of HS. Among these processes, the barrier function and immune response of intestinal epithelial cells have been identified as key pathological factors (Iba et al., 2025; Li et al., 2025; Ma et al., 2025). Notably, maintaining a balanced gut microbiota has been shown to enhance intestinal health and mitigate heat-induced intestinal damage (Lian et al., 2020).

Furthermore, our data suggest that APS has restorative effects on tissue integrity, which is essential for preventing such outcomes. Histological assessment and Western blot analysis of TJ proteins revealed that APS can mitigate the intestinal damage caused by HS. The protective effects of APS on the intestines suggest their ability to help maintain gut barrier function and reduce inflammation.

APS has multiple biological functions. Recently, an increasing number of studies have shown that APS may exhibit antitumorigenic potential (Kong et al., 2021; He et al., 2024). It has been proposed to increase the tumor response of and stabilize chemotherapy drugs while reducing their toxicity (Song et al., 2020; Yang et al., 2024). Moreover, APS is widely used in the treatment of cardiovascular diseases and diabetes; it can ameliorate insulin resistance and restore glucose homeostasis, improving insulin sensitivity in the liver and skeletal muscle in high-fat diet mice (Mao et al., 2009; Sun et al., 2019; Liu et al., 2024). APS has been reported to exhibit complex biological activities involved in the maintenance of intestinal barrier integrity, intestinal microbiota regulation, short-chain fatty acid production, and immune response regulation (Liang et al., 2024).

APS also has pronounced anti-inflammatory and immunomodulatory effects, such as enhancing natural killer cell and macrophage activity and increasing cytokine production and B cell proliferation (Clement-Kruzel et al., 2008; Liu et al., 2011; Li et al., 2022b). HS can trigger a systemic inflammatory response that leads to further complications. The ability of APS to reverse HS-induced changes in granulocyte counts and proportions in the blood, as well as reduce inflammatory cytokine levels in the intestines, demonstrates their anti-inflammatory properties. Another important finding was that APS reduced neutrophil infiltration and MPO^+^ cell counts. Although neutrophils are first-line defenders in the innate immune response, excessive activation can lead to tissue damage. APS appears to modulate the neutrophil response, which might prevent excessive inflammation and tissue damage during HS.

Heat acclimation is widely recognized as the best and most economical measure to prevent and protect against HS (Ashworth et al., 2020). Recent studies have demonstrated that the preventive administration of probiotics can reduce organ injuries against HS by regulating the gut microbiota (Peng et al., 2023; Li et al., 2024a; Xie et al., 2024). Therefore, we further examined the regulatory effect of pretreatment with APS on the gut microbiota. Similar to the previous study, there is a trend toward a decrease in the Firmicutes to Bacteroidetes ratio and an increase in the abundance of Proteobacteria (Zhong et al., 2022). The 16S rRNA sequence-based analysis of fecal samples indicated that APS treatment showed a better impact on gut microbiota via higher microbiota richness, which is beneficial to microbiota stability. Moreover, APS increased the abundance of beneficial genera such as Frisingicoccus and Lactococcus (Bagaria et al., 2024).

Based on PICRUSt2 and Tax4Fun predictions, bacterial community functions were significantly altered and mainly involved in metabolic modulation (e.g., vitamins B6 and B2). The vitamin B6 metabolism pathway has anti-inflammatory properties, which make it an interesting nutraceutical (Ueland et al., 2017). Vitamin B6 derivatives have been shown to reduce IL-33 expression, thereby limiting lung inflammation (Turnquist, 2023). A retrospective analysis showed that patients with Crohn’s disease frequently presented with reduced vitamin B6 levels occurred, which affected their intestinal flora (Feng et al., 2023). *In vitro* experiments also revealed that high-dose vitamin B6 demonstrated strong anti-inflammatory effects in LPS-stimulated monocytes (Mikkelsen et al., 2023). Recently, vitamin B6 has been found to possess anti-inflammatory and antiapoptotic effects through the inhibition of caspase-3 signaling pathway (Xu et al., 2024a).

Otherwise, vitamin B6 supplementation improves immune functions in vitamin B6-deficient humans and experimental animals. For example, dietary vitamin B6 intake modulates colonic inflammation in the IL-10-deficient mouse model of IBD (Selhub et al., 2013) and alleviates heat stress–induced intestinal barrier impairment by regulating the gut microbiota and metabolites in broilers (Ouyang et al., 2024). Through functional enrichment analysis of the microbiota, our study suggests that APS enhances vitamin B6 levels in the body. Subsequent quantification of serum vitamin B6 levels confirmed that APS pretreatment significantly increased vitamin B6 levels following HS exposure. This elevation may contribute to improved immune function, enhanced stress response, and strengthened intestinal barrier function. Furthermore, the riboflavin (vitamin B2) metabolism pathway was significantly enhanced after APS administration. In conclusion, both vitamin B6 and vitamin B2 may play an important role in the APS-evoked prevention of HS-induced inflammation.

Moreover, our network pharmacology analysis suggests that APS mitigates the detrimental effects of HS by inhibiting apoptosis. It is well-established that HS induces damage to the intestinal epithelial cell membrane and TJs, ultimately compromising intestinal integrity. This disruption increases intestinal permeability, which in turn leads to diminished growth performance and heightened morbidity and mortality (Xiao et al., 2013; Xiao et al., 2015; Xia et al., 2017; Zhang et al., 2023). Both *in vivo* experiments utilizing the mouse HS model and *in vitro* experiments with IEC-6 cells demonstrated that APS exerts an inhibitory effect on apoptosis by modulating the expression of Bak and cleaved caspase-3. The activation of Bax, Bak and Caspase-9 reflects the mitochondrial-mediated intrinsic apoptotic pathway, whereas caspase-3, a downstream executor, is a central player in both intrinsic and extrinsic apoptotic pathways (Boland et al., 2013; Van Opdenbosch and Lamkanfi, 2019).

In summary, APS may mitigate the progression of HS by attenuating intestinal inflammation, stabilizing gut microbiota and immune responses, and reducing epithelial apoptosis, underscoring its potential as a therapeutic agent. Given its diverse health-promoting properties, APS could be a key factor in managing HS effects. Nonetheless, further studies are warranted to elucidate its underlying mechanisms, optimal dosage, administration timing, and long-term efficacy in the treatment of HS and its associated complications.

## Data Availability

The bulk 16S rRNA sequencing data presented in the study are publicly available. This data can be found here: National Center for Biotechnology Information’s Sequence Read Archive (SRA), accession number PRJNA1331961.
